# Defining key design elements of registry-based randomised controlled trials: a scoping review

**DOI:** 10.1186/s13063-020-04459-z

**Published:** 2020-06-22

**Authors:** Bill Karanatsios, Khic-Houy Prang, Ebony Verbunt, Justin M. Yeung, Margaret Kelaher, Peter Gibbs

**Affiliations:** 1 Department of Surgery, The University of Melbourne, Parkville, VIC Australia; 2 Western Health Chronic Disease Alliance, Western Health, St Albans, VIC Australia; 3 Centre for Health Policy, The University of Melbourne, Parkville, VIC Australia; 4 Systems Biology and Personalised Medicine Division, The Walter and Eliza Hall Institute of Medical Research, Parkville, VIC Australia; 5 Department of Medical Biology, The University of Melbourne, Parkville, VIC Australia; 6 Department of Medical Oncology, Peter MacCallum Cancer Center, Parkville, VIC Australia

**Keywords:** Registry trials, Pragmatic trials, Real-world evidence, Registry

## Abstract

**Background:**

Traditional randomised controlled trials remain the gold standard for improving clinical care but they do have their limitations, including their associated high costs, high failure rate and limited external validity. An alternative methodology is the newly defined, prospective, registry-based randomised controlled trial (RRCT), where treatment and outcome data is collected in an existing registry. This scoping review explores the current literature regarding RRCTs to help identify the key design elements of RRCTs and the characteristics of clinical registries on which they are reliant on.

**Methods:**

A scoping review methodology conducted in accordance with the Joanna Briggs Institute guidelines was performed. Four databases were searched for articles published from inception to June 2018: Medline; Embase; the Cumulative Index to Nursing and Allied Health Literature and; Scopus. The search strategy included MeSH and text words related to RRCT.

**Results:**

We identified 2369 articles of which 75 were selected for full-text screening. Of these, only 17 articles satisfied our inclusion criteria. All studies were published between 1996 and 2017 and all were investigator-initiated. Study designs were mainly multi-site comparative/effectiveness studies incorporating the use of disease registries (*n* = 8), procedure registries (*n* = 8) and a health services registry (*n* = 1). The low cost, reduced administrative burden and enhanced external validity of RRCTs make them an attractive research methodology which can be used to address questions of public health importance. We identified that that there are variable definitions of what constituted a RRCT and that issues related to ethical conduct and data integrity, completeness, timeliness, validation and endpoint adjudication need to be carefully addressed.

**Conclusion:**

RRCTs potentially have an important role to play in informing best clinical practice and health policy. There are a number of issues that need to be addressed to optimise the utility of this approach, including establishing universally accepted criteria for the definition of a RRCT.

## Background

Randomised controlled trials (RCTs) are considered the gold standard for evaluating the effectiveness of medical interventions [1]. Despite their privileged status in the hierarchy of clinical evidence, the limitations of RCTs need to be acknowledged [2]. Traditional RCTs are complex and expensive to perform, they enrol a highly selected population, a high proportion fail to meet recruitment goals and they have limited external validity [3, 4], making it difficult to apply any learnings to the real-world patient population. Researchers are, therefore, turning their attention to alternative research methodologies in pursuit of more affordable and generalisable, high-quality clinical evidence [3, 5, 6].

One such alternative methodology is the registry-based randomised controlled trial (RRCT). RRCTs are generally considered under the broader umbrella of pragmatic trials [4, 7], although the definition of RRCTs is variable depending on how patients are recruited and whether clinical registries or routinely collected data (RCD) is used to capture outcomes [2, 8, 9]. One potential definition for a RRCT by Li et al. is a trial where eligible patients are identified and recruited from the registry, the patients’ existing baseline medical history is recorded in the registry and data related to the intervention and the outcomes are captured in the registry [2]. A randomisation module may also be incorporated within the registry [2, 10, 11].

Clinical registries can be disease, health services or product specific [12]. They collect clinical information for a specific area of interest and given the level of clinical detail that they capture, can support a variety of research questions. In contrast, RCD such as electronic health records (EHR) and administrative/claims data, support clinical care and administrative or billing activities and, as such, there are limits to how this data can be used to support research. RCD may lack detailed information on clinical indications, patient characteristics, type of treatment, and outcome events and may also be less structured (e.g. free text) [13].

RRCTs are best suited for testing hypotheses involving pharmaceutical interventions, devices, and any other intervention already available in the real-world clinical setting but where there is variable implementation or uncertainty regarding optimal treatment combination, sequencing or duration, or where multiple standard-of-care options are available. Hard endpoints, such as overall survival, are preferred. Given that the adverse events associated with the intervention under study are already well-established, less detail regarding adverse events needs to be captured [14, 15]. The benefits of a RRCT to assess the comparative effectiveness of treatments in a real-world setting have been demonstrated through the pioneering and landmark Thrombus Aspiration during ST-Segment-Elevation Myocardial Infarction (TASTE) [16] and the SAFE-PCI for Women studies [17].

Despite these benefits, there are several challenges in the design and implementation of RRCTs that need to be addressed, including: data quality, regulatory and ethical issues, adjudication of study outcomes, choice of methodology and study design, and operational challenges emanating from the type of clinical registry and RCD being used [2, 8, 18]. The diversity of elements that may underpin a RRCT study adds further complexity to this subject matter. As the RRCT is a new and evolving clinical trial methodology, the need to reach consensus on a set of elements, that should comprise and define RRCTs, is of high importance. To date, one review of RRCTs has been conducted but the identification and recruitment of eligible patients from a clinical registry was not a prerequisite [8]. As such, it is possible that the review included post-trial extension of RCTs to assess long-term outcomes using clinical registries.

The aims of this scoping review are to describe the literature covering RCTs embedded within clinical registries, and to identify the key design elements of RRCTs and the characteristics of clinical registries that enabled them to support RRCTs. We elected to use the RRCT definition of Li et al. [2] as we consider clinical registries as playing a pivotal role in the conduct of RRCTs. We acknowledge the variability of data quality and completeness across registries. However, clinical registries are more likely to contain greater depth of clinical information and exhibit a greater level of data validation than RCD [19]. Supplementary RCD may help strengthen the internal validity of RRCTs by reducing loss to follow-up and non-random missing data. As the RRCT methodology is still in its infancy, it is opportune that an attempt is made to better elucidate the essential elements required for a study to be considered a RRCT, and the characteristics that clinical registries must have in order to adequately support RRCTs.

## Methods

The scoping review methodology was selected to map the literature in this emerging area. The scoping review was conducted in accordance with Joanna Briggs Institute methodology guidelines for conducting scoping studies [20] and reported using the Preferred Reporting Items for Systematic reviews and Meta-Analyses extension for Scoping Reviews (PRISMA-ScR) [21]. The methodology draws on the Arksey and O’Malley framework [22]: (1) identifying the research question; (2) identifying relevant studies; (3) study selection; (4) charting the data; and (5) collating, summarising and reporting the results. The objectives, inclusion criteria and methods for this scoping review were specified in advance and documented in an unpublished protocol which can be made available upon request. The protocol was strictly followed with one minor deviation from the search strategy, in which articles were also identified from a RRCT review article [8].

### Data sources and searches

Four databases were searched for articles published from inception to June 2018: Medline, Embase; the Cumulative Index to Nursing and Allied Health Literature (CINHAL); and Scopus. Articles were also screened from a review on RRCTs [8]. The reference list of all identified articles was searched for additional studies (hand search). The search strategy was conducted with the assistance of a librarian. The search strategy included MeSH and text words related to RRCT (Appendix 1).

### Study selection

#### Inclusion and exclusion criteria

Articles were included if they met all of the following criteria: (1) limited to clinical registry trials that involved selection of participants from a registry or simultaneously enrolled participants into the registry and a trial; (2) randomisation of participants to an intervention or a control group; and (3) collection of at least one outcome measure from a clinical registry. Articles were excluded if they met any of the following criteria: (1) RRCT protocols; (2) registries of clinical trials; (3) trials using electronic health records and administrative databases to select participants; (4) non-randomised registry trials; (5) observational studies/retrospective cohort studies using registries; (6) registry-based follow up of RCT studies; (7) studies that did not derive any outcomes from a clinical registry and (8) published in languages other than English.

The search results from each database were imported into Endnote X8 and duplicates were excluded. Two authors (BK and KP) independently screened the titles and abstracts for relevance and then assessed the eligibility of the full-text articles. Discrepancies between authors were discussed between them and if they remained unresolved, a third author made the final decision.

### Data extraction and synthesis

A standardised data charting form to record key information was created based on the protocol. The following information was extracted from the articles by two authors (BK and KP) independently: authors; year of publication; country; study design; aim; population; sample size; registry name; randomisation; intervention; trial duration and follow-up; and outcomes. Additional information pertaining to the clinical registries was extracted: purpose; time-period coverage; population coverage; consent; funding; variables; validity and reliability. Descriptive information of studies was reported. A narrative synthesis of the findings from the studies was conducted, with a focus on summarising the key design elements of a RRCT (recruitment, randomisation and outcomes) and characteristics of clinical registries (operating infrastructure and data quality) that enable them to support a RRCT.

## Results

### Study selection

**Fig. 1:**
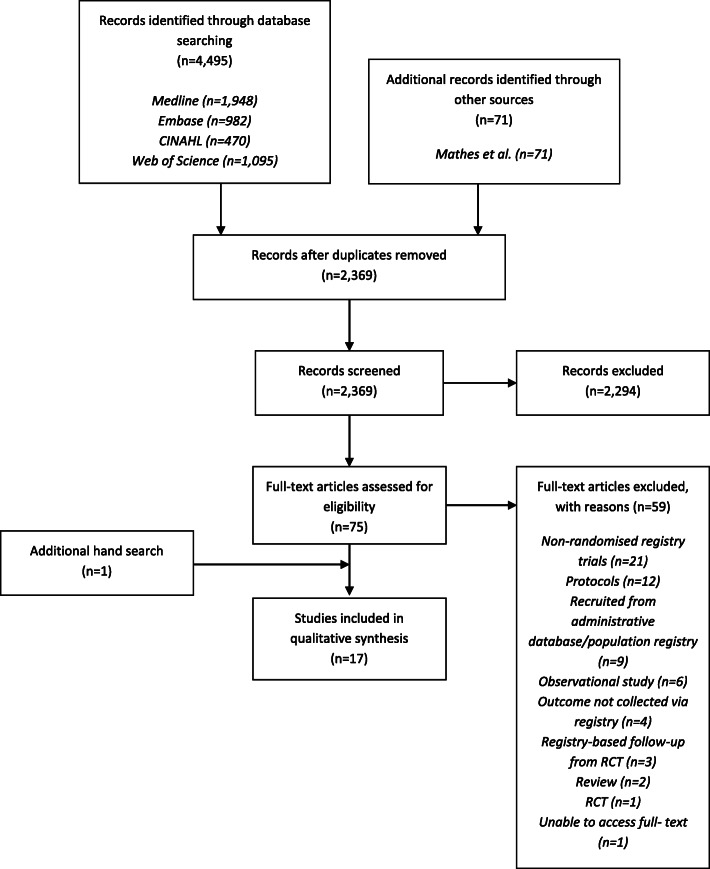
Flow diagram for retrieval of articles

We identified 2369 articles from four databases and one review article (Fig. 1). Following title and abstract screening, we excluded 2294 articles, leaving 75 articles for full-text screening. Following full-text screening, we excluded 59 articles of which: 21 were non-randomised registry trials; 12 were protocols; nine involved recruitment of participants from either administrative databases or population registries; six were observational studies; four did not collect any outcome from a registry; three were a registry-based follow-up RCT; two were a review; one was a RCT that did not involve a registry; and one we were unable to obtain full text. The remaining 16 articles satisfied the review inclusion criteria. An additional article was included via hand search. We included 17 articles in our synthesis.

### Study characteristics

The characteristics of the 17 studies that satisfied the inclusion criteria of our review are described in Table 1. All studies were published between 1996 and 2017. Studies were predominantly conducted in the USA (*n* = 8), followed by Sweden (*n* = 4), Denmark (*n* = 2), Australia (*n* = 1), Italy (*n* = 1) and The Netherlands (*n* = 1). All studies were investigator-initiated, supported by a combination of research grants and cooperative agreements. In four studies, investigators also received unrestricted grants from commercial entities [6, 16, 23, 24]. Study designs were predominantly multi-site comparative/effectiveness studies, incorporating the use of disease registries (*n* = 8), procedure registries (*n* = 8) and a health services registry (i.e. ICU) (*n* = 1). Two studies were a registry-based follow-up of RRCT studies in percutaneous intervention and breast cancer conducted a year [24] and 12 years earlier [25], respectively. The total sample size across all studies was 59,330. The sample size per study ranged from 112 to 10,175. Study populations included patients with heart conditions (*n* = 8), unvaccinated children (*n* = 6), cancer patients (*n* = 2) and patients in intensive care units (ICUs) (*n* = 1). Interventions comprised patient reminders (*n* = 7), surgical procedures (*n* = 4), non-surgical procedures (*n* = 2), drug treatments (*n* = 2) and performance feedback (*n* = 2).

**Table 1:** Registry-based randomised controlled trial (RRCT) characteristics

	***N*** studies
**Population size**	
≤ 100	1
> 100–500	2
> 500–1500	2
> 1500–5000	7
> 5000	5
**Trial duration**	
≤ 12 months	6
> 12–18 months	3
> 18–36 months	7
> 60 months	1
**Outcome measures**	
Intervention uptake	7
Mortality	7
Health outcomes	8
Performance outcomes	1
**Follow-up period**	
≤ 3 days	1
2–4 weeks	2
2–5 months	4
6–11 months	5
1–2 years	2
> 3 years	1
Not applicable	2
**Loss to follow-up**	
0%	5
1–5%	3
6–10%	1
> 10%	1
Not reported	5
Not applicable	2

### RRCT study features

#### Recruitment

Ten studies used clinical registries to identify eligible participants for enrolment in RRCTs. Of these, six studies identified eligible children from immunisation registries [26–31]; two studies identified eligible participants from an Australian state-based cancer registry [32], and a registry of percutaneous aortic valve replacement procedures (REPLACE) [33], respectively; and two studies identified potential ICUs and hospitals from the Dutch National Intensive Care Evaluation (NICE) registry [34] and the National Cardiovascular Data Registry (NCDR) ACTION Registry® GWTG^TM^ [35], respectively. Seven studies simultaneously enrolled participants in the clinical registries and a RRCT. That is, participants who were enrolled in the clinical registries were also prospectively screened for RRCT eligibility. Of these, four studies utilised the Swedish Web System for Enhancement and Development of Evidence-based Care in Heart Disease Evaluated According to Recommended Therapy (SWEDEHEART) registry [6, 16, 24, 36]. Other registries included the National Cardiovascular Data Registry CathPCI [17], the Western Denmark Heart Registry [23] and the Danish Breast Cancer Cooperate Group (DBCG) registry [25].

#### Randomisation

Randomisation was commonly performed at the individual level, except for two studies where randomisation occurred at the hospital [35] and department (ICU) level [34]. Eight studies used a computer-generated code or random number for randomisation of participants into arms [23, 26–28, 30, 32–34]. In five studies, randomisation was performed within the clinical registry using an online randomisation module [6, 16, 17, 24, 36]. Four studies did not explicitly describe their randomisation processes, merely noting that participants were randomised [25, 29, 31, 35].

#### Interventions

Studies commonly involved two arms, except for two studies which consisted of three [31] and four arms [30], respectively. The interventions were comprised of clinical and non-clinical interventions. Clinical intervention studies included four surgical procedures [16, 17, 23, 24], two non-surgical procedures [33, 36] and two drug treatments [6, 25]. Surgical and non-surgical procedure studies investigated procedures/devices where the short- and long-term efficacy appeared to be uncertain in sub-groups of the population. For example, Frobert and Lagerqvist [16] and Lagerqvist and Frobert [24] examined the effect of thrombus aspiration on mortality within 30 days and 1 year among participants with ST-segment-elevation myocardial infarction. Rao and Hess [17] compared the impact of radial with femoral artery access on bleeding/vascular complications within 72 h of the procedure among women undergoing percutaneous coronary intervention (PCI). Similarly, the pharmacological studies investigated drug treatments where the relative short- and long-term efficacy were unclear. Erlinge and Omerovic [6] compared the effect of bivalirudin versus heparin monotherapy in reducing mortality within 180 days among participants with acute coronary syndrome treated with PCI. Kristensen and Ejlertsen [25] investigated the long-term impact of adjuvant tamoxifen and local radiotherapy versus local radiotherapy alone on femoral fractures among postmenopausal women with breast cancer.

Non-clinical studies included seven studies where the interventions were patient reminders [26–32] and two performance feedback surveys [34, 35]. Patient-reminder interventions generally compared various modes of reminders (e.g. letter, telephone) with standard/no reminder (control group) on immunisation rates. The performance feedback studies compared targeted/activated feedback with standard feedback/benchmark reports on patient outcomes.

#### Outcomes and loss to follow-up

Primary outcomes in clinical intervention studies focussed on well-defined clinical endpoints: all-cause mortality at 30 days [16] and within 1 year [24, 36]; a composite of death from any cause, myocardial infarction, or major bleeding during 180 days of follow-up [6]; a composite of cardiac death, myocardial infarction, or target-lesion revascularisation within 1 year [23]; bleeding/vascular complications requiring intervention occurring within 72 h of the procedure/hospital discharge [17]; incidence of acute kidney injury occurring within 72 h after the procedure [33]; and occurrence of fractures [25].

Secondary outcomes in clinical intervention studies included: rehospitalisation [16, 24, 36]; target-vessel/lesion-revascularisation [16, 23, 24]; and complications and length of stay [16]. Primary outcomes in non-clinical interventions were: immunisation doses as recorded in the clinical registry [26–29, 31]; improvement in the overall composite of all metrics (i.e. acute measures, discharge, excess dosing and reperfusion measures) [35]; ICU length of stay [34]; and response rate [32].

Primary outcomes were predominantly collected from the clinical registry. In some studies, additional outcomes were collected via data linkage with other registries and/or administrative datasets [6, 16, 23–25, 31, 36] and case report forms [33].

Trial duration across all studies ranged from 2 months to 2 years and 9 months and follow-up period ranged from 72 h to 12 years. Loss to follow-up for clinical intervention studies were minimal: four studies had no patient loss to follow-up [16, 24, 25, 33]; three had < 2% loss to follow-up [6, 17, 23]; and one had 6% loss to follow-up [36]. For non-clinical intervention studies, loss to follow-up was rarely reported or was not applicable to the study design.

Only three studies provided any commentary on the cost-effectiveness of their study [17, 28, 30] Two of the studies pertained to vaccination reminders and associated labour costs [28, 30] whilst the third study provided a cursory cost comparison of a cardiology intervention RRCT against the potential cost of a similar conventional RCT [17].

#### Registry features

Table 2 describes the characteristics of the clinical registries. The studies involved 13 clinical registries: disease registries (*n* = 6); procedure(s) registries (*n* = 6); and a health services registry (*n* = 1). Several studies utilised the same clinical registries: SWEDEHEART registry (*n* = 4) [6, 16, 24, 36]; the Michigan Care Improvement Registry (*n* = 2) [27, 28]; the NCDR (*n* = 2) [17, 35]. The primary purpose of all clinical registries was to monitor and improve patient care. Clinical registries covered a wide range of populations: children; patients with myocardial infarction; and patients with cancer. Population coverage ranged from one hospital in Italy [33] to 86% of all ICUs in The Netherlands [34].

**Table 2:** Characteristics of clinical registries

	***N*** studies
**Time-period coverage**	
Reported	8
Not reported	5
**Population coverage**	
Reported	13
Not reported	0
**Consent stated**	
Yes	9
No	4
**Funding**	
Reported	10
Not reported	3
**Data validity**	
Reported	10
Not reported	3

Period coverage was reported in seven clinical registries. Most of the clinical registries were established in the 1990s, with the earliest registry, the DBCG in Denmark developed in 1977. The most recently established registry was the SWEDEHEART in Sweden which was developed in 2009.

Variables collected in the clinical registries were dependent on the type of clinical registry, with the most common variables collected being patient demographics, medical history and clinical outcomes. Data quality was briefly reported in seven clinical registries. The approach to data-quality assurance varied across the studies and included: validation rules (e.g. out-of-range values); random review of data entered in the clinical registry with hospital records; and data record completeness. Data validity was noted in 10 studies and the method of achievement varied from study to study and involved fully automated processes utilising algorithms or manual spot-checking of registry data against the medical record.

Funding sources for clinical registries were not disclosed, except for three clinical registries. The NCDR ACTION Registry® GWTG^TM^, SWEDEHEART and the NCDR CathPCI were supported by multiple sources of funding including government agencies, non-profit/charitable-fundraising organisations and pharmaceutical companies. The approaches to patient consent (e.g. waiver of consent, opt in or opt out) to have data included in the clinical registries were not described.

## Discussion

Our scoping review yielded a total of 17 studies. Of these, eight were in cardiology, six in immunisation, two in oncology and one in critical care. The clinical interventions ranged across comparative studies of drugs, devices or procedures. These interventions were mostly confined to the cardiology and oncology studies. For the non-clinical interventions, such as the immunisation and critical care studies, the intervention was a vaccination reminder and performance feedback, respectively. The majority of the studies were multi-centre, involved large sample sizes and included long follow-up periods with minimal loss to follow-up.

Most clinical registries were already relatively well-established, having been operational for a number of years prior to their utilisation in a RRCT. Furthermore, most of the registries included in our review were either national registries or at minimum State- or district-based. The Nordic countries exhibited the most comprehensive national registries that facilitated the enrolment of patients onto the registry upon confirmation of their disease, with the Swedish national cardiac registry – SWEDEHEART being an online registry that supported four RRCTs.

Data validity and data integrity of registries are critical elements in realising the full potential and scope of RRCTs [14]. In our review, 10 studies provided information on registry-data validity. Only three studies commented on missing data [29, 31, 36], whilst 14 studies remained silent on this matter. In countries with well-established national registries, data validation appears robust with minimal data missing. This, coupled with recruitment of large patient cohorts, enables RRCTs using such registries to not only have strong external validity but to also afford good internal validity. This confers them properties more akin to RCTs and makes them a viable alternative for obtaining high-quality clinical evidence. Whereas, RRCTs that are reliant on registries that are not robust or not subject to adequate data-validation processes may produce findings which cannot reliably inform clinical practice or health policy.

Therefore, data validation of registries and other data sources used in a RRCT is imperative if RRCTs are to move up the hierarchy of clinical evidence to position themselves as a valid alternative to the conventional RCT. Before embarking on the conduct of a RRCT, researchers should have a clear appreciation of the data collected in the registry(ies) and any supplementary RCD that their study will need to rely on, and the quality and validation of all data sources. Understanding how data is collected in the clinical registry is important to avoid misinterpreting results that are the consequence of data-entry error or bias. Based on this, the study design should be such that it adequately compensates for any deficiencies that such data sources may present, with researchers fully aware of these limitations in advance and actively looking to appropriately address them [2].

The ability of RRCTs to identify and recruit more effectively than conventional RCTs, due to the availability of searchable clinical information in the clinical registry enabling screening for eligible patients, is well-documented [36, 37]. In the TASTE trial, 76.9% of all eligible patients were randomised within 2 years and 9 months [2]. However, where participant registration onto the registry is not timely, or the registry has limited catchment coverage, this can pose a number of challenges. This was most evident in the vaccination studies, resulting in the non -recording of patients who sought their intervention outside of the registry catchment area. Invitations for participation were also issued to individuals who were no longer eligible [26, 31]. This becomes even more important for clinical intervention studies conducted within acute settings, as enrolment onto the registry and randomisation must be close to real time or at least concurrent with commencement of an intervention, so that the conduct of the study does not impact on the provision of best care. This was evident in several cardiology studies [16, 17].

The embedding of a randomisation module into the registry expedites the recruitment and randomisation of patients into a RRCT. The online nature of the SWEDEHEART registry allows for immediate enrolment of patients into the registry upon hospital admission and identification of the need for a PCI. As this registry is a nationally supported and funded initiative, capture of clinical data and validation of outcome measures is well-managed. This results in the conduct of RRCTs, such as the TASTE trial, providing high-quality clinical evidence; thus, demonstrating that RRCTs are a viable alternative to the more expensive standard RCT. It is apparent that for registries to accommodate RRCTs within acute clinical settings, an online registry platform that provides real-time registry enrolment of potential participants, is essential. Furthermore, the embedding of a randomisation module within a registry may help to address the intervention time constraints in such settings.

All of the six clinical intervention studies were open-label, multi-centre RRCTs. These studies examined two standard-of-care interventions in clinical settings [6, 16, 17, 23, 33, 36]. Only one of these six studies provided an explanation as to why a blinded design was not pursued. In this study, the choice for an open-label design was justified by feasibility and ethical considerations and the unavailability of a suitable sham comparator [36]. The remaining five interventional studies did not comment on the reasons for their open-label design. However, as reduced cost is a driving force behind conducting RRCTs we surmise that the cost of making the study double blinded may have been prohibitive. It is appreciated that there are increased costs associated with the manufacture and provision of a placebo control aimed to mimic in appearance or application the active intervention. It is also acknowledged that blinding is not always practically possible when two different standards of care are being compared. Most studies did acknowledge that the open-label design of the study was a study limitation, potentially biasing study outcomes. A double-blinded study design was not applicable for the non-clinical intervention studies.

Given the broad inclusion criteria of a RRCT, adequately powered trials can potentially be conducted at a single site, dependent on the type of event being investigated and the population size the site services. Multi-centre studies allow for the recruitment of a much larger number of participants into a trial, and this is necessary where either the disease being investigated and/or the outcome event is rare [3]. It is acknowledged that despite the large participant numbers and broad inclusion criteria, RRCTs may be subject to reduced external validity if the intended study population is geographically or socioeconomically restricted. For example, some of the vaccination-reminder studies identified this as a potential limitation of their study, as some of the populations targeted were of a restricted socioeconomic status [26, 29, 30].

Ethical and governance considerations are aspects of RRCTs that remain active areas of work. Given the breadth of research activity that can fall under the classification of a RRCT and the varied jurisdictional requirements, there can be no ‘one rule fits all’ approach. Six studies that involved both ethical approval and at a minimum oral consent involved a clinical intervention whereby randomisation determined the standard-of-care intervention to be provided. Consent was obtained in most studies prior to randomisation, and the inability to provide consent was a study exclusion criterion. For studies that had time constraints in relation to the delivery of an intervention, oral consent was deemed acceptable. This was then followed by written consent at a later and more appropriate time. In contrast, seven studies did not mention that they had ethical approval and made no reference to any form of consent from the participants. These studies were either vaccination participation invitation letters or a quality improvement study, whereby ethical approval and, by default, active participant consent were not considered to be necessary [26–29, 31, 34, 35]. Two studies that did not directly involve a clinical intervention [25, 32] obtained ethical approval but did not involve consent for participation. Consent for participation on a registry and/or RRCT is an area that requires further exploration and the approach will be informed by the ethical and governance requirements of the jurisdiction in which the registry resides and where the trial is being conducted.

Central adjudication of study endpoints, along with dedicated follow-up and systematic monitoring in RRCTs is critical to ensure the quality of the data related to the study outcome measures [38]. In our review, most studies involving a clinical intervention involved hard endpoints, such as mortality, in addition to other intervention-related study outcome measures. Five studies commented on the adjudication of their study outcome measures [6, 16, 17, 23, 36]. Of these, two confirmed a blinded endpoint adjudication process [6, 23]. One study confirmed that there was no adjudication of their study outcomes but relied on strict diagnostic indicators for defining the primary endpoint [33]. It is appreciated that hard endpoints, such as death from any cause, do not require adjudication [36]. For studies that utilised the SWEDEHEART registry, most relied on that registry for adjudication of their study outcomes, with no further study-specific adjudication of outcomes being made. The lack of central adjudication of study outcome measures in RRCTs has been a well-acknowledged limitation and becomes even more critical when RRCTs are multi-centre or, particularly, if there is intention for multinational involvement. Furthermore, the lack of adjudication, coupled with lower quality or missing data does necessitate more complex statistical methods to be utilised, which may inadvertently intimidate the reader [36].

In most of the clinical intervention studies, the hard endpoints included death from any cause, and the required outcomes were collected via data linkage of clinical registries with administrative population/claims data. This was most evident in the studies conducted in the Nordic countries where unique patient identification numbers facilitate complete tracking of patients across registries and other sources of databases; thus, allowing near complete follow-up of all participants [2]. Furthermore, the use of a registry in a RRCT allows for the long-term follow-up of participants. In our review, we identified two follow-up studies of a RRCT. The first was the 1-year post-TASTE follow-up study and the second, a 12-year post-follow-up study of a retrospective RRCT looking at bone fractures in women treated with tamoxifen for breast cancer[25]. In the TASTE study, which had more than 7244 participants, there was no single patient lost to follow-up; again highlighting the advantages of well-established registries and the ability to easily link to supplementary datasets using a unique patient identification number [9].

For RRCTs to provide high-quality clinical evidence, the challenges of outcome adjudication and data integrity and quality need to be addressed through the establishment of registries and/or datasets that have integrated quality assurance processes embedded into their administration. The use of supplementary datasets in conjunction with a registry can help to minimise the occurrence of missing or inaccurate data by facilitating data triangulation and providing a better understanding around data validity and integrity. However, in countries without a unique patient identifier, data linkage to enable the collection of primary/secondary outcomes may not always be possible and other means to collect such outcomes must be explored. In such circumstances, researchers would need to rely on data linkage across a number of records through appropriate data-linkage software. In Victoria, Australia, the Centre for Victorian Data Linkage (CVDL) utilises a deterministic data-linkage method whereby records across a number of registries and datasets are determined to belong to the one person on the basis of returning an exact match for a set of fields [39]. Probabilistic data linkage is also an option with obvious inherent limitations. However, the pursuit of enhanced RRCT internal validity must be carefully balanced so that the administrative and economic benefits that make RRCTs a viable alternative to conventional RCTs are not progressively eroded.

RRCTs are considered to be a cost-effective way of obtaining quality clinical evidence compared to conventional RCTs. Three studies provided a cost-benefit assessment of conducting a RRCT [17, 28, 30]. The SAFE-PCI study cost approximately US$5 million to conduct due to the utilisation of the NCDR CathPCI Registry for streamlined data collection and randomisation. A comparably sized trial not underpinned around a registry would have cost considerably more [17]. For the TASTE trial, costs were estimated at 10% or less of a conventional RCT [38, 40]. The increased cost-effectiveness of RRCTs can be attributed to RRCTs obtaining their outcome data from registries or RCD, reducing requirements for follow-up visits, monitoring and audits. Furthermore, as RRCTs utilise and rely on existing infrastructure and human resources, the need for new equipment and training of staff is limited. Given that 9–14% of a RCT’s total cost can be attributed to site monitoring, it is not surprising that the reduction or even elimination of many of the activities that comprise the essential compliance aspects of a RCT would result in substantive cost savings [9].

Despite the cost benefits of RRCTs, they are not yet readily afforded commercial support in comparison to conventional RCTs. Of all the studies, two cardiology studies had unrestricted commercial support in addition to academic grant funding. The support of RRCTs by industry should be a welcomed involvement, as it will allow for the conduct of adequately funded studies and the introduction of investment that is essential in building the requisite infrastructure and processes required to help overcome the challenges of RRCTs and to enhance their internal validity. Furthermore, industry participation in RRCT studies would result in RCTs not necessarily underpinned on commercial imperatives but ones that address questions of public health importance. Ideally, to alleviate any concerns in relation to undue industry influence on the topic of investigation, namely study design and result reporting, any industry support in investigator-initiated RRCTs should be prefaced around the provision of unrestricted grants or like funding. The prospect of industry embracing RRCTs in lieu of conventional RCTs does not appear to be an imminent prospect, but one which, over time, will evolve and increase in occurrence as industry better appreciates how RRCTs can complement RCTs, and the academic establishment learns to work alongside commercial entities in a synergistic and complementary manner and feels comfortable accepting and pursuing such arrangements. Future research is warranted to investigate industry’s perspective of RRCTs and to further explore the barriers that have limited their involvement to date.

To our knowledge, there are currently no guidelines for the reporting of a RRCT and this presents several challenges. It is acknowledged that RRCTs should be underpinned by the Consolidated Standards of Reporting Trials Statement (CONSORT Statement). RRCTs should provide information on the quality of the registry itself, particularly around elements of data quality which should include, but not be limited to: accuracy, completeness, timeliness, population coverage and study endpoint adjudication. The reporting of consent into the registry, and subsequently into a RRCT, need to be improved, as does the financial disclosure for both the registry and the RRCT. An extension of the CONSORT Statement for RCTs using cohorts and routinely collected health data is currently underway to improve the quality of reporting [41].

### Limitations

Whilst the search that we conducted was extensive and included a wide range of relevant electronic databases, it did not include studies in languages other than English and of the grey literature. Given that RRCTs are a novel research design, the absence of indexing terms for RRCTs increases the possibility that some studies may not have been captured by our search terms. Furthermore, a lack of a precise definition for a RRCT makes it challenging to ascertain the research activity in this space and its impact. Depending on the criteria used to define a RRCT, the number of studies captured will vary considerably. This is evident in the review conducted by Mathes and Buehn [8] which used a broader definition to define RRCT, resulting in 71 studies being included. RRCTs underpinned around RCD, such as electronic health records and administrative claims data, were excluded from our review, but we recognise the role of RCD in supplementing information required in the conduct of a RRCT and helping to address some of their inherent limitations. A combination of RCD and actively collected data, such as a clinical registry, may make a trial more feasible [9]. Future research is warranted to assess the feasibility of using RCD in RCTs. Furthermore, most studies only briefly described the quality of their registries and provided limited information about ethical approval and the consent process. It is unclear whether this represents reporting bias or merely highlights the lack of emphasis placed on these aspects, given that there is an inherent expectation of lower data quality and integrity for such trials compared to conventional RCTs. We also acknowledge that this review did not explicitly explore qualitative barriers and enablers to the use of RRCTs. We believe that further research in this area is warranted to help increase the implementation of RRCTs.

## Conclusion

RRCTs have an important role to play in informing best clinical practice and health policy. Their low cost, reduced administrative burden and enhanced external validity make them an attractive research methodology to be used to address questions of public health importance. However, for RRCTs to be considered a viable alternative to a RCT in certain clinical settings, the issues of data integrity, completeness, timeliness, validation and adjudication of endpoints need to be carefully addressed. It would be our recommendation that RRCTs should be registered as is the case for RCTs and that RRCTs should be underpinned by the CONSORT Statement. Our review also highlights the variable definitions being used for a RRCT and reinforces the need for universally accepted criteria to be established, such that the current broad criteria that are in use do not dilute the influence and impact of studies that carry the real hallmarks of a RRCT.

## Data Availability

All data generated or analysed during this study is included in this published article.

## Appendix 1

### Search strategy

registry-based randomised clinical trial* or

registry-based randomized clinical trial* or

registry-based randomised control* clinical trial* or

registry-based randomized control* clinical trial* or

registry-based randomised control* trial*or

registry-based randomized control* trial* or

registry embedded clinical trial* or

registry trial* or

registry-based randomized trial* or

registry-based randomised trial* or

pragmatic trial* or

pragmatic clinical trial*

## Appendix 2

**Table 3:** Data extraction of included studies (*N* = 17)

Authors	Year	Country	Study design and aim	Population and sample size	Registry name	Randomisation	Intervention Trial duration Follow-up Loss to follow-up	Outcomes Type Data linkage
Alexander et al. [35]	2011	USA	Cluster randomised controlled trial To test a strategy of specific and targeted performance feedback vs. standard feedback for its ability to better facilitate quality improvement	Hospitals in The National Cardiovascular Data Registry (NCDR®) ACTION Registry® – GWTG™ *N* = 149 Intervention *n* = 76 Control *n* = 73	The National Cardiovascular Data Registry (NCDR®) ACTION Registry® – GWTG™	Randomisation was stratified by baseline quality performance score, academic status, and cardiac services (hospitals with cardiac surgery vs. other).	Targeted feedback vs. standard feedback. Trial duration = 18 months Follow-up = not applicable Loss to follow-up = not applicable	Performance measures and quality metrics based on the 2008 American College of Cardiology/American Heart Association Performance Measures for myocardial infarction care and ACTION Registry® – GWTG^TM^ metrics - improvement in the overall composite of all metrics and composite of the 3-site-specific selected metrics - trends in patient outcomes including in-hospital bleeding and mortality
Barbanti et al. [33]	2015	Italy	Investigator-driven, single-centre prospective, open-label, registry-based randomised trial To investigate the effect of the RenalGuard System on prevention of acute kidney injury (AKI) in patients undergoing transcatheter aortic valve replacement (TAVR)	All consecutive patients with symptomatic severe aortic stenosis undergoing TAVR *N* = 112 Intervention *n* = 56 Control *n* = 56	Registry of percutaneous aortic valve replacement (the REPLACE registry)	Patients were 1:1 randomly assigned. Randomisation was obtained with computer-generated codes, which were sealed in sequentially numbered envelopes	RenalGuard vs. standard management. Trial duration = 11 months Follow-up = 72 h Loss to follow-up = 0 patient	The incidence of AKI occurring within the first 72 h after the procedure - trial-specific information, including renal outcomes of interest not obtained as part of the registry, were collected using additional case report form pages
Daley et al. [26]	2002	USA	Randomised controlled trial To assess the efficacy of letter/telephone recall for immunisation with pneumococcal conjugate vaccine (PCV7) in an economically disadvantaged urban population	All children aged 6 weeks to 22 months, identified from an immunisation registry database *N* = 1420 Intervention *n* = 610 control *n* = 624	Immunisation registry database	Immunisation registry and Microsoft Excel 97 were used to randomly assign subjects to study arms	Letter/telephone recall vs. control Trial duration = 2 months Follow-up = 2 months Loss to follow-up = not reported	Receipt of 1 or more doses of PCV7 during the 2-month study period, as recorded in the immunisation registry
Dombkowski et al. [27]	2012	USA	A registry-based randomised trial To assess the feasibility and effectiveness of using a state-wide immunisation information system (IIS) for seasonal influenza vaccine reminders from local health departments (LHDs) targeting children with high-risk conditions	Children aged 24–60 months with high-risk conditions living in 3 county LHD jurisdictions with primarily English-speaking households, identified using the MCIR *N* = 3618 Intervention *n* = 1810 Control *n* = 1808	Michigan Care Improvement Registry (MCIR	Within each LHD jurisdiction, children were sorted by a random number, with half assigned to the intervention (reminder) group and half to the control (no reminder) group	Reminder notices vs. no reminder notices Trial duration = 4 months Follow-up = 4 months Loss to follow-up = 729 children	Effectiveness was based on the outcome of 1 or more seasonal influenza vaccination doses being entered into MCIR - feasibility of reminder delivery was evaluated through letters returned by the USPS as undeliverable and through the (NCOA) Link results
Dombkowski et al. [28]	2014	USA	A registry-based randomised trial To assess the relative effectiveness of centralised reminder/recall strategies targeting age-specific vaccination milestones among children in urban areas during June 2008–June 2009	Children aged 7 or 19 months and not up to date for at least 1 dose and children turning age 12 months, regardless of their vaccination status. Eligible children identified via MCIR *N* = 10,175 7-month recall *n* = 2072 12-month reminder *n* = 3502 19-month recall *n* = 4601	MCIR	Children for reminder/recall were randomised to either the notification (intervention) or no notification group (control), using an automated group assignment process	Notification vs. no notification Trial duration = 12 months Follow-up = 60 days Loss to follow-up = not reported	MCIR-recorded immunisation activity (administration of Z1 new dose, entry of Z1 historic dose, entry of immunisation waiver) within 60 days following each notification cycle - the completeness of immunisation activity following the date of notification and the timing of immunisation activity
Erlinge et al. [6]	2017	Sweden	Investigator-initiated, multi-centre, randomised, registry-based, open-label clinical trial To investigate whether the use of bivalirudin would result in a lower rate of the composite of death from any cause, myocardial infarction and major bleeding events than heparin monotherapy (without planned use of glycoprotein IIb/IIIa inhibitors) among patients with either ST-segment-elevation myocardial infarction (STEMI) or non-STEMI (NSTEMI) who were undergoing PCI predominantly with the use of radial-artery access and who were receiving treatment with potent P2Y12 inhibitors	Patients admitted to the hospital with a diagnosis of STEMI or NSTEMI and for whom urgent PCI was planned *N* = 6006 STEMI *n* = 3005 NSTEMI *n* = 3001	Swedish Web System for Enhancement and Development of Evidence-based Care in Heart Disease Evaluated According to Recommended Therapies (SWEDEHEART registry)	Randomisation was performed in a 1:1 ratio in permuted blocks, with the use of a computer-generated list, with stratification according to type of myocardial infarction (STEMI or NSTEMI) and hospital. Randomisation conducted via the online Swedish Coronary Angiography and Angioplasty Registry (SCAAR), which is a component of the SWEDEHEART registry	Open-label fashion either intravenously administered bivalirudin (the Medicines Company) vs. intraarterial unfractionated heparin (LEO Pharma) Trial duration = 26 months Follow-up = 180 days Loss to follow-up = 68 patients	A composite of death from any cause, myocardial infarction, or major bleeding during 180 days of follow-up obtained from the Swedish National Population Registry - answers to trial-specific questions were collected in a separate trial specific module embedded in the SWEDEHEART online questionnaire
Frobert et al. [16]	2013	Sweden	Investigator-initiated, multi-centre, prospective, randomised, registry-based, controlled, open-label clinical trial To evaluate whether thrombus aspiration reduces mortality	Patients with chest pain suggestive of myocardial ischemia for at least 30 min before hospital admission, if the time from the onset of symptoms to hospital admission was less than 24 h, and if an electrocardiogram (ECG) showed new ST-segment-elevation or left bundle-branch block *N* = 7244 Intervention *n* = 3621 Control *n* = 3623	SWEDEHEART registry	Patients were randomly assigned, in a 1:1 ratio, and randomisation was performed by means of an online randomisation module within SCAAR	Manual thrombus aspiration followed by PCI vs. PCI only Trial duration = 2 years and 9 months Follow-up = 30 days Loss to follow-up = 0 patient	All-cause mortality at 30 days with data on mortality obtained from the national population registry - 30-day rates of hospitalisation for recurrent myocardial infarction, stent thrombosis, target-vessel revascularisation, target- lesion revascularisation and the composite of all-cause mortality or recurrent myocardial infarction obtained from the SWEDEHEART registry and the national discharge registry - complications of PCI, stroke or neurological complications, heart failure, and length of stay in the hospital obtained from the registries
Hall et al. [32]	2013	Australia	Randomised controlled trial To assess the effectiveness of an ‘enhanced’ invitation letter in increasing participation in an Australian cancer registry-based study and assess the representativeness of the study sample	Cancer survivors who had haematological cancer, including leukaemias, lymphomas and myelomas, and aged between 18 and 80 years, identified from 1 Australian state-based cancer registry *N* = 800 Intervention *n* = 400 Control *n* = 400	Australian state-based cancer registry	Registry staff used random number allocation to randomise survivors into 1 of 2 groups.	Modified invitation letter, incorporating content and design characteristics recommended to improve written communication vs. standard invitation letter Trial duration = 4 weeks Follow-up = not applicable Loss to follow-up = not applicable	Response rate and representativeness of the study sample collected in the cancer registry
Hofmann et al. [36]	2017	Sweden	Multi-centre, parallel-group, open-label, registry-based, randomised, controlled trial To evaluate the effect of oxygen therapy on all-cause mortality at 1 year among patients with suspected myocardial infarction who did not have hypoxemia at baseline	Patients with suspected myocardial infarction and an oxygen saturation of 90% or higher *N* = 6629 Intervention *n* = 3311 Control *n* = 3318	SWEDEHEART, Swedish National Population Registry and Swedish National Inpatient and Outpatient Registries	Unrestricted 1:1 randomisation, using a computer-generated list with the use of an online randomisation module embedded in SWEDEHEART	Oxygen (at 6 l per min for 6 to 12 h delivered through an open face mask) vs. ambient air Trial duration = 2 years and 8 months Follow-up = 12 months Loss to follow-up = 403 patients	Rehospitalisation with heart failure and cardiovascular death obtained from SWEDEHEART and the Swedish National Population Registry – including data on mortality and the vital status of all Swedish citizens
Irigoyen et al. [31]	2006	USA	Randomised controlled trial To conduct a randomised controlled trial to assess the relative effectiveness of 2 serial registry reminder protocols at an inner-city practice network	Children aged 6 weeks to 15 months, who had made at least 1 visit to the network, and were due or late for a diphtheria, tetanus and pertussis (DTaP) dose. Eligible children were identified weekly using EzVac *N* = 1662 Continuous reminder *n* = 549 Limited reminder *n* = 552 Control *n* = 561	EzVac (hospital immunisation registry)	Randomisation was carried out weekly	Continuous reminders vs. limited reminders vs. control group Trial duration = 6 weeks Follow-up = 6 months Loss to follow-up = not reported	Received any subsequent immunisation and age-appropriate, up-to-date status at 3 and 6 months tracked with EzVac - additional immunisations in CIR records for each child not-up-to-date status
Jensen et al. [23]	2016	Denmark	A large-scale, registry-based randomised, multi-centre, single-blind, 2-arm, non-inferiority trial To compare 2 biodegradable polymer drug-eluting stents: the thin-strut cobalt-chromium sirolimus-eluting Orsiro stent and the stainless-steel biolimus-eluting Nobori stent in an all-comer patient population	Patients ≤ 18 years old, chronic stable coronary artery disease or acute coronary syndromes, and at least 1 coronary lesion with > 50% diameter stenosis, requiring treatment with a DES *N* = 2525 Intervention *n* = 1261 Control *n* = 1264	The Civil Registration System; the National Registry of Patients; The Western Denmark Heart Registry; The National Registry of Causes of Deaths	Randomly allocated 1:1 block randomisation by centre. An independent organisation computer generated the allocation sequence, stratified by sex and presence of diabetes mellitus. Patients were assigned to treatment through a web-based Trial Partner randomisation system	The biodegradable polymer sirolimus-eluting stent vs. the biodegradable polymer biolimus-eluting stent Trial duration = 15 months Follow-up = 12 months Loss to follow-up = 2 patients	Data on mortality, hospital admission, coronary angiography, repeat percutaneous coronary intervention (PCI), and coronary-artery-bypass surgery were obtained from the following national Danish administrative and health care registries: the Civil Registration System; the Western Denmark Heart Registry; and the Danish National Registry of Patients, which maintains records on all hospitalisations in Denmark
Kempe et al. [29]	2005	USA	Randomised controlled trial To assess the maximal influenza immunisation rates that could be achieved for healthy young children in a private practice setting, to evaluate the efficacy of registry-based reminder/recall for influenza vaccination, and to describe methods used by private practices to implement the recommendations	All healthy children aged 6–21 months of age from 5 paediatric practices, identified from an immunisation registry and billing database *N* = 5193 Intervention *n* = 2595 Control *n* = 2598	Immunisation registry	Random allocation of subjects stratified according to practice site	3 reminder/recall letters generated by the immunisation registry vs. usual care Trial duration = 6 months Follow-up = 6 months Loss to follow-up = not reported	Receipt of > 1 influenza immunisation, recorded in either the immunisation registry or billing data
Kristensen et al. [25]	1996	Denmark	Retrospective analysis of a prospective, randomised trial based on computerised registries of occurrence of breast cancer and admissions to hospitals To investigate the occurrence of femoral fractures from a randomised study in postmenopausal women treated with adjuvant tamoxifen and local radiotherapy vs. local radiotherapy alone	Postmenopausal breast cancer women *N* = 1716 Intervention *n* = 868 Control *n* = 848	Danish National Registry of Patients (DNRP) and Danish Breast Cancer Cooperative Group (DBCG) registry	Allocation to treatment was randomised	Adjuvant tamoxifen and local radiotherapy vs. local radiotherapy alone. Trial duration = 5 years and 3 months Follow-up = 12 years Loss to follow-up = 0 patient	Occurrence of femoral fractures as recorded in the DNRP registry
Lagerqvist et al. [24]	2014	Sweden	Multi-centre, prospective, randomised, controlled, open-label clinical trial To evaluate clinical outcomes at 1 year after thrombus aspiration	Patients who had planned treatment of acute ST-segment-elevation myocardial infarction (STEMI) *N* = 7244 Intervention *n =* 3621 Control *n* = 3623	SCAAR, National Population Registries and SWEDEHEART	Patients were randomly assigned, in a 1:1 ratio. Randomisation was performed by means of an online randomisation module within the SCAAR database	Routine thrombus aspiration before PCI vs. PCI alone Trial duration = 2 years and 9 months Follow-up = 12 months Loss to follow-up = 0 patient	Death from any cause, rehospitalisation for myocardial infarction, stent thrombosis, target-vessel revascularisation and target-lesion revascularisation, collected from the registry - events of a post-hoc-defined composite of death from any cause, rehospitalisation for myocardial infarction, or stent thrombosis
LeBaron et al. [30]	2004	USA	Randomised controlled trial To test whether large-scale reminder recall could meaningfully raise low inner-city immunisation rates and to compare the impact and costs of different forms of reminder recall: in-person telephone calls, mailings and home visits vs. computer-generated telephone calls and mailings	Children aged 24–60 months with high-risk conditions living in 3 county LHD jurisdictions with primarily English-speaking households, identified in an immunisation registry as receiving health care in the public sector *N* = 3050 Combination *n* = 764 Outreach *n* = 760 Autodialer *n* = 763 Control *n* = 763	Metro Atlanta Team for Child Health (MATCH) immunisation registry	Participants were assigned by computer-generated random numbers to 1 of 4 groups	1 of 4 groups: autodialer only, outreach only, combination (outreach for children not vaccinated after completion of the autodialer protocol), vs. control (no interventions beyond normal clinic procedure, which in certain cases involved non-automated postcard recall systems). Trial duration = 24 months Follow-up = 24 months Loss to follow-up = not reported	Vaccinations from all providers, public and private, received by study children by 24 months of age and present in the MATCH registry
Rao et al. [17]	2014	USA	Investigator-initiated, multi-centre trial, prospective, open-label, registry-based, randomised controlled clinical trial To determine the effect of radial access on outcomes in women undergoing percutaneous coronary intervention (PCI)	Women undergoing cardiac catheterisation or percutaneous coronary intervention (PCI) *N* = 1787 Intervention *n* = 893 Control *n* = 894	National Cardiovascular Data Registry’s CathPCI Registry	Patients were randomised 1:1 ratio. Randomisation was performed via an online randomisation module incorporated into the registry trial database	Radial access vs. femoral arterial access Trial duration = 22 months Follow-up = 72 h and 30 days Loss to follow-up = 27 patients	The primary efficacy endpoint was bleeding or vascular complications requiring intervention occurring within 72 h of the procedure or by hospital discharge, whichever came first; as identified in the CathPCI Registry - the primary feasibility endpoint was access site crossover
Van der Veer et al. [34]	2013	The Netherlands	Cluster randomised trial To assess the impact of applying a multifaceted activating performance feedback strategy on intensive care patient outcomes compared with passively receiving benchmark reports	Intensive care units (ICUs) participating in the Dutch National Intensive Care Evaluation (NICE) registry *N* = 30 ICUs Intervention *n* = 15 ICUs Control *n* = 15 ICUs *N* = 25,552 admissions	The Dutch National Intensive Care Evaluation (NICE) registry	Randomised ICUs (i.e. clusters), because the intervention was targeted at the facility rather than patient level. ICUs were randomised according to their size and ability to collect data as determined from prior feasibility study. Randomisation of ICUs was conducted by a dedicated software, blinding those enrolling and assigning the ICUs. Participants were not blinded neither were those involved in providing the strategy	Activating performance feedback strategy vs. benchmark reports Trial duration = 16 months Follow-up = 14 months Loss to follow-up = 0 ICUs	ICU length of stay, as recorded in the NICE data

## Appendix 3

**Table 4:** Characteristics of clinical registries (*N* = 13)

Studies	Type	Name	Purpose	Time-period coverage	Population coverage	Consent	Funding	Variables	Validity/reliability
Alexander et al. 2011 [35]	Disease registry	The National Cardiovascular Data Registry (NCDR) ACTION Registry® – GWTG^TM^	Platform for hospitals to measure and improve their myocardial infarction care and to advance quality improvement efforts	Not reported	Detailed clinical information on > 150,000 existing patients with either ST-elevation or non-ST-elevation myocardial infarction (STEMI and NSTEMI)	Not reported	Initiative of the American College of Cardiology Foundation and the American Heart Association, with partnering support from The Society of Chest Pain Centres, The Society of Hospital Medicine and The American College of Emergency Physicians. The registry is sponsored by Bristol-Myers Squibb/Sanofi Pharmaceuticals	Patient demographics, presenting features, pre-hospital and in-hospital therapies, timing of care delivery, laboratory tests, procedure use, and in-hospital patient outcomes	Data entered via a secure password-protected, web-based server system with programmed frontend logic and range checks to optimise data quality at the time of data entry
Barbanti et al. 2015 [33]	Procedure registry	Registry of Percutaneous Aortic Valve Replacement (the REPLACE registry)	Created to monitor the institutional procedural, acute and long-term outcomes of transcatheter aortic valve replacement	Not reported	Ferrarotto Hospital in Catania, Italy	Yes	Not reported	Patient demographics, medical history, concomitant medications, procedure details, and in-hospital clinical outcomes are routinely entered in the registry’s electronic data collection system using standardised case report forms	Not reported
Daley et al. 2002 [26]	Procedure registry	Immunisation registry	Administered vaccines are entered into the registry daily, and it operates in accordance with nationally recommended standards for immunisation registries	Began in May 1998	The Children’s Hospital, Denver, Colorado, USA	Not reported	Not reported	Age of child, immunisation status and uptake of new vaccines (such as PCV7)	Validation of registry immunisation data by performing a chart review of 40 randomly selected records. The registry error rate was 8%, calculated as the percentage of immunisations documented in the medical records but not in the registry. The registry duplicate record rate was less than 1%
Kempe et al. 2005 [29]	Procedure registry	Immunisation registry	Immunisation records for all children < 72 months of age in the participating practices were entered into an existing regional immunisation registry 15–24 months before the present study	Not reported	47% of children 0 to 6 years of age in Colorado	Not reported	Not reported	Immunisation data from medical records and billing data	Quality assessment data for the 5 practices, demonstrated an overall completeness rate of 97.4% (children in the practice who were in the registry) and an error rate of 7.2% (immunisation not in the registry or incorrect date)
Dombkowski et al. 2012 [27]; Dombkowski et al. 2014 [28]	Procedure registry	Michigan Care Improvement Registry (MCIR)	MCIR is used widely by public and private providers throughout Michigan; state law requires that all vaccination doses administered to children aged less than 20 years be entered into MCIR	Not reported	Children aged less than 20 years in Michigan	Not reported	CDC Cooperative Agreement	In addition to tracking individual vaccination doses, MCIR has extensive assessment, reporting, and reminder/recall notification capabilities based on recommendations of the National Vaccine Advisory Committee	2012 Validation of addresses conducted retrospectively using US Postal Services 2014 Not reported on; Data validation/reliability
Frobert et al. 2013 [16]; Lagerqvist et al. 2014 [24]; Erlinge et al. 2017 [6]; Hofmann et al. 2017^a^ [36]	Disease registry	Swedish Web System for Enhancement and Development of Evidence-based Care in Heart Disease Evaluated According to Recommended Therapy (SWEDEHEART registry)	Merging of the national registry of acute cardiac care (RIKSHIA), the Swedish Coronary Angiography and Angioplasty Registry (SCAAR), the Swedish heart surgery registry and the national registry of secondary prevention (SEPHIA). The main purpose of the registry is to support the improvement of care and evidence-based development of therapy of coronary artery disease by providing continuous information on care needs, therapy and results of therapy and changes within a hospital as well as in comparison to other hospitals	Began in December 2009	Patients admitted to hospital because of symptoms suggestive of an acute coronary syndrome (ACS), and patients undergoing coronary angiography/angioplasty or heart surgery from all 29 Swedish and 1 Icelandic coronary intervention centres	Yes for all 4 studies	The Swedish Association of Local Authorities and Regions (the public health care provider), and is supported by the Swedish Heart Association, the National Board of Health and Welfare and the Swedish Heart and Lung Foundation. Participating hospitals are not reimbursed by the registry and costs of local data entry are borne by their internal budget	106 variables and include patient demographics, admission logistics, risk factors, past medical history, medical treatment prior to admission, electrocardiographic changes, biochemical markers, other clinical features and investigations, medical treatment in hospital, interventions, hospital outcome, discharge diagnoses and discharge-medications	Uppsala Clinical Research Centre provides manuals, education and technical advice, including a telephone help desk for all users of the registry. The system has error-checking routines for range and consistency. Definitions are easily available when data are entered. To ensure the correctness of the data entered a monitor visits about 20 hospitals each year and compares data entered into the SWEDEHEART with the information in the patients’ records from 30 to 40 randomly chosen patients in each hospital
Hall et al. 2013 [32]	Disease registry	Australian state-based cancer registry	It is a legal requirement that all cancer diagnoses are notified to the relevant cancer registry.	Not reported	1 Australian state	No consent required	Not reported	Age, gender, cancer type, year of diagnosis, postcode and other demographic and disease characteristics were collected from the cancer registry	Not reported
Irigoyen et al. 2006 [31]	Procedure registry	EzVac	To consolidate immunisation records for a hospital health care system	Began in 2000	All children born or receiving care at the hospital or affiliated practices	Not reported	Funding provided by U66/CCU 212961 National Immunisation Program	Immunisation	Internal validity of EzVac assessed by comparing immunisation in EzVac with paper medical records. External validity of EzVac assessed by comparing immunisations from EzVac with CIR
Jensen et al. 2016^b^ [23]	Disease registry	The Western Denmark Heart Registry (WDHR)	To monitor and improve the quality of cardiac intervention in Western Denmark and to allow for clinical and health-service research	Began in 1999	All adult (≥ 15 years) patients in Western Denmark referred for cardiac intervention, i.e. invasive procedures (coronary angiography or percutaneous coronary intervention), cardiac surgery (predominantly valve surgery and coronary artery bypass grafting), and from 2008 also computed tomography coronary angiography	Yes	Collaborative effort by Western Denmark’s 3 major cardiac centres (Aarhus University Hospital-Skejby, Odense University Hospital and Aarhus University Hospital-Aalborg). The participating centres own the WDHR and finance its operation through annual membership fees set according to hospital size	For each procedure, physicians report administrative data, including dates of referral, admission, operation and discharge; and clinical data, including medical history, procedure data, lesion-data, complications, and research study enrolments. Depending on the procedure type, 50 to 150 variables are registered for each procedure	The data quality is ensured by automatic validation rules at data entry combined with systematic validation procedures and random spot-checks after entry
Kristensen et al. 1996^c^ [25]	Disease registry	The Danish Breast Cancer Cooperative Group Registry	To improve the prognosis in breast cancer	Began in 1977	90% of all women with breast cancer in Denmark	Not reported	Until 1982, financed from private sources: The Danish Health and Medicines Authority and the Finsen Institute. Thereafter, financed from counties and from 2007 Danish regions	Characteristics of the primary tumour, of surgery, radiotherapy and systemic therapies, and of follow-up reported on specific forms from the departments	Queries are sent to the departments if reporting is missing according to the guidelines indicated on the forms, or if the database receives forms from 1 discipline (for instance, pathology), but not from the corresponding discipline (for instance, surgery or oncology). The completeness has improved to more than 95%
LeBaron et al. 2004 [30]	Procedure registry	Metro Atlanta Team for Child Health (MATCH) immunisation registry	To improve immunisation rates (providing provider measurement and feedback)	Began in 1993	Atlanta metropolitan area: the public health clinics of DeKalb County (the other county that includes part of Atlanta), the area’s federally qualified community health centres, the 2 major private paediatric hospitals with their outpatient and satellite centres, the 2 major academic medical facilities, the major Roman Catholic hospital with its outpatient and outreach facilities, and a number of large private practices	Not reported	Not reported	Vaccination doses, race and sex	Not reported
Rao et al. 2014^d^ [17]	Disease registry	The National Cardiovascular Data Registry (CathPCI)	To assist health care providers and institutions in documenting their processes and outcomes of care in the cardiac catheterisation laboratory	Began in 1997	85% of the cardiac catheterisation laboratories in the United States	Yes	Co-sponsored by the American College of Cardiology and the Society for Cardiovascular Angiography and Intervention	Demographics, medical history, concomitant medications, procedure details, in-hospital clinical outcomes	‘Formally validated’ – no further information provided
Van der Veer et al. 2013 [34]	Health services registry	The Dutch National Intensive Care Evaluation (NICE) registry	To systematically and continuously monitor and improve ICU performance by reporting and benchmarking quality indicators	Began in 1996	86% of all Dutch ICUs	Yes	Not reported	11 structure, process and outcome indicators	Data collected through the NICE registry which has its own inbuilt infrastructure validation systems

^a^Additional information extracted from supplementary file [36]

^b^Additional information extracted from cited paper [42]

^c^Additional information extracted from Christiansen, Ejlertsen [43]

^d^Additional information extracted from Dehmer, Weaver [44]

## Abbreviations

CVDL: Centre for Victorian Data Linkage
DBCG: Danish Breast Cancer Cooperate Group
EHR: Electronic health records
ICU: Intensive care unit
NCDR: National Cardiovascular Data Registry
PCI: Percutaneous coronary intervention
RCD: Routinely collected data
RCT: Randomised controlled trial
RRCT: Registry randomised controlled trial
SWEDEHEART: Swedish Web System for Enhancement and Development of Evidence-based Care in Heart Disease Evaluated According to Recommended Therapy
TASTE: Thrombus Aspiration during ST-Segment-Elevation Myocardial Infarction
VCCC: Victorian Comprehensive Cancer Centre

## References

[1] Fitzpatrick T, Perrier L, Tricco AC, Straus SE, Juni P, Zwarenstein M (2017). Protocol for a scoping review of post-trial extensions of randomised controlled trials using individually linked administrative and registry data. BMJ Open.

[2] Li G, Sajobi TT, Menon BK, Korngut L, Lowerison M, James M (2016). Registry-based randomized controlled trials—what are the advantages, challenges, and areas for future research?. J Clin Epidemiol.

[3] Bergqvist D, Bjorck M, Sawe J, Troeng T (2007). Randomized trials or population-based registries. Eur J Vasc Endovasc Surg.

[4] Califf RM, Sugarman J (2015). Exploring the ethical and regulatory issues in pragmatic clinical trials. Clin Trials.

[5] Barnish MS, Turner S (2017). The value of pragmatic and observational studies in health care and public health. Pragmatic Observational Res.

[6] Erlinge D, Omerovic E, Frobert O, Linder R, Danielewicz M, Hamid M (2017). Bivalirudin versus heparin monotherapy in myocardial infarction. N Engl J Med.

[7] Concannon TW, Guise J-M, Dolor RJ, Meissner P, Tunis S, Krishnan JA, et al. A national strategy to develop pragmatic clinical trials infrastructure. CTS Journal. 2014;7(2):164–71. 10.1111/cts.12143 PMC4126802 24472114

[8] Mathes T, Buehn S, Prengel P, Pieper D (2018). Registry-based randomized controlled trials merged the strength of randomized controlled trails and observational studies and give rise to more pragmatic trials. J Clin Epidemiol.

[9] Mc Cord KA, Al-Shahi Salman R, Treweek S, Gardner H, Strech D, Whiteley W (2018). Routinely collected data for randomized trials: promises, barriers, and implications. Trials [Electronic Resource].

[10] James S, Frobert O, Lagerqvist B (2012). Cardiovascular registries: a novel platform for randomised clinical trials. Heart.

[11] Jones WS, Roe MT, Antman EM, Pletcher MJ, Harrington RA, Rothman RL (2016). The changing landscape of randomized clinical trials in cardiovascular disease. J Am Coll Cardiol.

[12] Gliklich RE, Dreyer NA, Leavy MB (2014). Registries for evaluating patient outcomes: a user’s guide.

[13] Meinecke AK, Welsing P, Kafatos G, Burke D, Trelle S, Kubin M (2017). Series: pragmatic trials and real world evidence: Paper 8. Data collection and management. J Clin Epidemiol.

[14] Liu JB, D’Angelica MI, Ko CY (2017). The Randomized Registry Trial: Two Birds, One Stone. Ann Surg.

[15] Foroughi S, Wong HL, Gately L, Lee M, Simons K, Tie J, et al. Re-inventing the randomized controlled trial in medical oncology: The registry-based trial. Asia-Pac J Clin Oncol. 2018;14:365–73. 10.1111/ajco.12992. 10.1111/ajco.12992 29947051

[16] Frobert O, Lagerqvist B, Olivecrona GK, Omerovic E, Gudnason T, Maeng M (2013). Thrombus aspiration during ST-segment elevation myocardial infarction. N Engl J Med.

[17] Rao SV, Hess CN, Barham B, Aberle LH, Anstrom KJ, Patel TB (2014). A registry-based randomized trial comparing radial and femoral approaches in women undergoing percutaneous coronary intervention: the SAFE-PCI for Women (Study of Access Site for Enhancement of PCI for Women) trial. JACC Cardiovasc Interv.

[18] Lauer MS, D’Agostino RB (2013). The Randomized Registry Trial — The Next Disruptive Technology in Clinical Research?. N Engl J Med.

[19] Maier B, Wagner K. Comparing routine administrative data with registry data for assessing quality of hospital care in patients with myocardial infarction using deterministic record linkage. BMC Health Serv Res. 2016;16(605). 10.1186/s12913-016-1840-5. 10.1186/s12913-016-1840-5 PMC5073420 27769288

[20] The Joanna Briggs Institute (2015). The Joanna Briggs Institute reviewers' manual 2015: Methodology for JBI scoping reviews.

[21] Tricco ACea (2018). PRISMA extension for scoping reviews (PRISMA-ScR): checklist and explanation. Ann Intern Med.

[22] Arksey H, O’Malley L (2005). Scoping studies: towards a methodological framework. Int J Soc Res Methodol.

[23] Jensen LO, Thayssen P, Maeng M, Ravkilde J, Krusell LR, Raungaard B, et al. Randomized Comparison of a biodegradable polymer ultrathin strut sirolimus-eluting stent with a biodegradable polymer biolimus-eluting stent in patients treated with percutaneous coronary intervention. The SORT OUT VII Trial. Circ Cardiovasc Interv. 2016;9(7):e003610. 10.1161/CIRCINTERVENTIONS.115.003610. 10.1161/CIRCINTERVENTIONS.115.003610 27412869

[24] Lagerqvist B, Frobert O, Olivecrona GK, Gudnason T, Maeng M, Alstrom P (2014). Outcomes 1 year after thrombus aspiration for myocardial infarction. N Engl J Med.

[25] Kristensen B, Ejlertsen B, Mouridsen HT, Andersen KW, Lauritzen JB (1996). Femoral fractures in postmenopausal breast cancer patients treated with adjuvant tamoxifen. Breast Cancer Res Treat.

[26] Daley MF, Steiner JF, Brayden RM, Xu S, Morrison S, Kempe A (2002). Immunization registry-based recall for a new vaccine. Ambul Pediatr.

[27] Dombkowski KJ, Harrington LB, Dong S, Clark SJ (2012). Seasonal influenza vaccination reminders for children with high-risk conditions: a registry-based randomized trial. Am J Prev Med.

[28] Dombkowski KJ, Costello LE, Harrington LB, Dong S, Kolasa M, Clark SJ (2014). Age-specific strategies for immunization reminders and recalls: a registry-based randomized trial. Am J Prev Med.

[29] Kempe A, Daley MF, Barrow J, Allred N, Hester N, Beaty BL (2005). Implementation of universal influenza immunization recommendations for healthy young children: results of a randomized, controlled trial with registry-based recall. Pediatrics..

[30] LeBaron CW, Starnes DM, Rask KJ (2004). The impact of reminder-recall interventions on low vaccination coverage in an inner-city population. Arch Pediatr Adolesc Med.

[31] Irigoyen MM, Findley S, Wang D, Chen S, Chimkin F, Pena O (2006). et al. Challenges and successes of immunization registry reminders at inner-city practices. Ambul Pediatr.

[32] Hall AE, Sanson-Fisher RW, Lynagh MC, Threlfall T, D’Este CA (2013). Format and readability of an enhanced invitation letter did not affect participation rates in a cancer registry-based study: a randomized controlled trial. J Clin Epidemiol.

[33] Barbanti M, Gulino S, Capranzano P, Imme S, Sgroi C, Tamburino C (2015). Acute kidney injury with the RenalGuard system in patients undergoing transcatheter aortic valve replacement. The PROTECT-TAVI Trial (PROphylactic effecT of furosEmide-induCed diuresis with matched isotonic intravenous hydraTion in Transcatheter Aortic Valve Implantation). JACC Cardiovasc Interv.

[34] van der Veer SN, de Vos ML, van der Voort PH, Peek N, Abu-Hanna A, Westert GP (2013). Effect of a multifaceted performance feedback strategy on length of stay compared with benchmark reports alone: a cluster randomized trial in intensive care. Crit Care Med.

[35] Alexander KP, Wang TY, Li S, Lytle BL, Slattery LE, Calhoun S (2011). Randomized trial of targeted performance feedback to facilitate quality improvement. Circ Cardiovasc Qual Outcomes.

[36] Hofmann R, James SK, Jernberg T, Lindahl B, Erlinge D, Witt N (2017). Oxygen therapy in suspected acute myocardial infarction. N Engl J Med.

[37] Ieva F, Gale CP, Sharples LD (2014). Contemporary roles of registries in clinical cardiology: when do we need randomized trials?. Expert Rev Cardiovasc Ther.

[38] James S, Rao S, Granger C (2015). Registry-based randomized clinical trials— A new clinical trial paradigm. Nat Rev Cardiol.

[39] Department of Health & Human Services. The Centre for Victorian Data Linkage 2018. Available from: https://www2.health.vic.gov.au/about/reporting-planning-data/the-centre-for-victorian-data-linkage.

[40] Ashrafi R, Hussain H, Brisk R, Boardman L, Weston C (2014). Clinical disease registries in acute myocardial infarction. World J Cardiol.

[41] Kwakkenbos L, Juszczak E, Hemkens LG, Sampson M, Fröbert O, Relton C (2018). Protocol for the development of a CONSORT extension for RCTs using cohorts and routinely collected health data. Res Integrity Peer Rev.

[42] Schmidt M, Maeng M, Jakobsen C-J, Madsen M, Thuesen L, Nielsen PH (2010). Existing data sources for clinical epidemiology: the Western Denmark Heart Registry. Clin Epidemiol.

[43] Christiansen P, Ejlertsen B, Jensen M-B, Mouridsen H (2016). Danish Breast Cancer Cooperative Group. Clin Epidemiol.

[44] Dehmer GJ, Weaver D, Roe MT, Milford-Beland S, Fitzgerald S, Hermann A (2012). A contemporary view of diagnostic cardiac catheterization and percutaneous coronary intervention in the United States: a report from the CathPCI Registry of the National Cardiovascular Data Registry, 2010 through June 2011. J Am Coll Cardiol.

